# Aniline-induced production of aniline-containing polyketides and related bicyclic polyketides by the Yellow River wetland-derived fungus *Talaromyces funiculosus*

**DOI:** 10.3389/fmicb.2023.1200680

**Published:** 2023-05-17

**Authors:** Zhenhui Wang, Zhanlin Li, Chao Niu, Lanping Yang, Yangyang Zhai, Dehai Li, Guangwei Wu, Zhenzhen Zhang, Xueqian He

**Affiliations:** ^1^School of Medicine, Henan Polytechnic University, Jiaozuo, China; ^2^Key Laboratory of Marine Drugs, Chinese Ministry of Education, School of Medicine and Pharmacy, Ocean University of China, Qingdao, China; ^3^College of Chemical Engineering, Nanjing Forestry University, Nanjing, China

**Keywords:** aniline, tetrahydronaphthone polyketides, NMR, antimicrobial activity, *Talaromyces funiculosus*

## Abstract

**Introduction and Methods:**

Silencing gene activation can effectively enrich the diversity of fungal secondary metabolites.

**Results and Discussion:**

Cultivation of the Yellow River wetland-derived fungus *Talaromyces funiculosus* HPU-Y01 with aniline led to the isolation of one new aniline-containing polyketide tanicutone A (**1**), two new bicyclic polyketides tanicutones B-C (**2**–**3**), a new related trienoic acid 8-methyldeca-2,4,6-trienoic acid (**5**), and a known compound **4**. The planar structures and configurations of **1**–**5** were determined by NMR, MS, and ECD calculations. Compound **2** featured a key aldehyde group and showed promising inhibitory activity against *Vibrio parahaemolyticus* with a minimum inhibitory concentration (MIC) value of 0.17 μg/mL. This is a rare report of aniline-induced fungal production of tetrahydronaphthone polyketides.

## Introduction

In-depth bioinformatics analysis of fungi has indicated that most of the biosynthetic genes for secondary metabolites are unexpressed under general laboratory conditions (Li et al., 2020; Lyu et al., 2020). Activation of these gene clusters to obtain novel carbon skeleton for drug leads attracted extensive attention (Bauman et al., 2021). Chemical epigenetic modification is an effective method to activate cryptic genes by simply adding DNA methyltransferase inhibitors or histone deacetylase inhibitors to the medium of microorganisms (Cichewicz, 2010). Significantly, suberoylanilide hydroxamic acid (SAHA) was one of the most popular histone deacetylase inhibitors (He et al., 2018; Guo et al., 2021; Li et al., 2022). However, recent reports showed SAHA could be metabolized by fungi into aniline, and then formed aniline-containing metabolites, such as chalaniline A (Adpressa et al., 2017), cladosins J-K (Zhang et al., 2018), and talaroenamine B (Zhang et al., 2021). These successes indicate that aniline could be a promising exogenous-added chemical incorporated by microorganisms into the active biosynthetic intermediates to the discovery of new “unnatural” product scaffolds.

**Figure 1:**
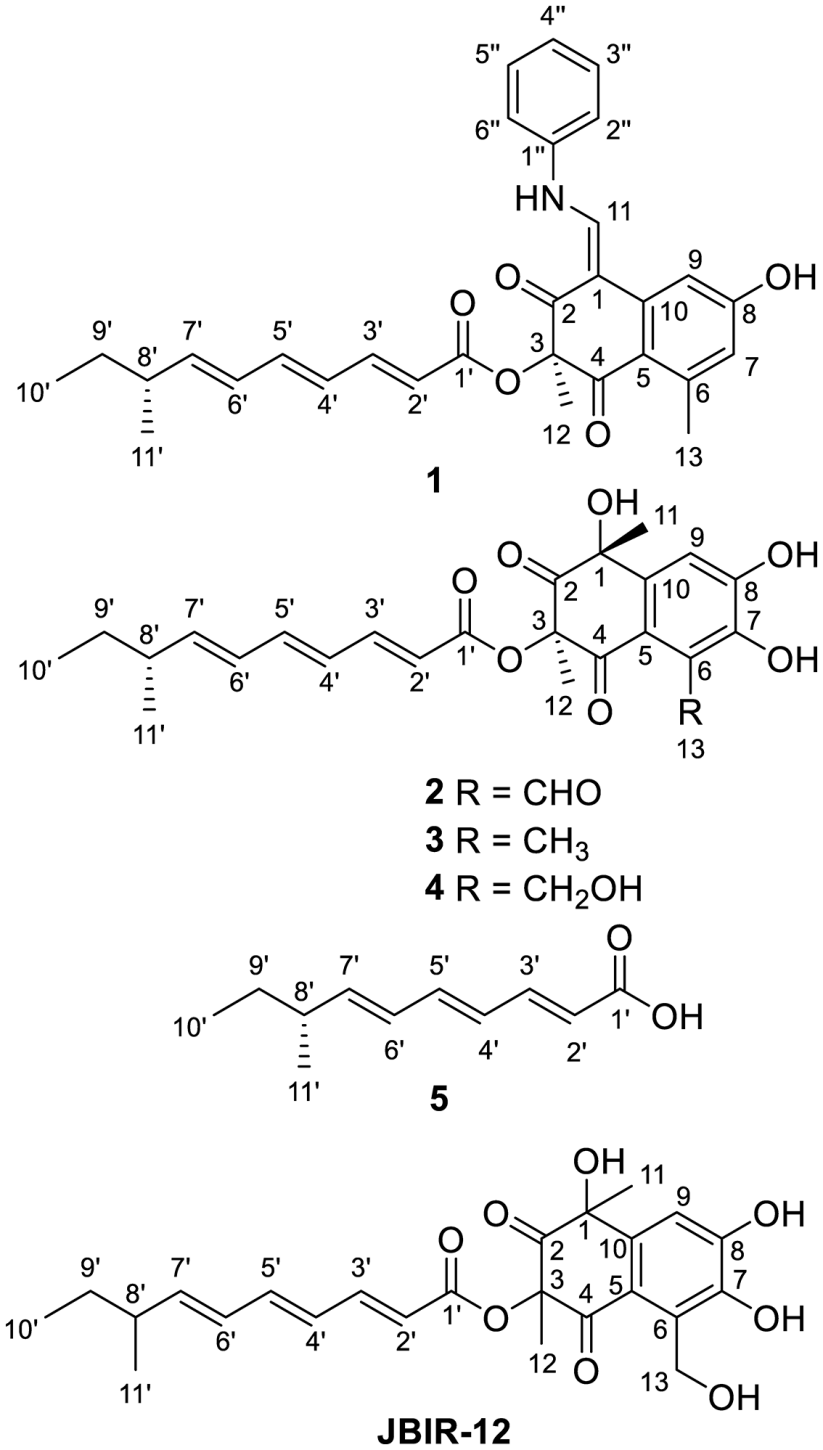
Structures of compounds **1**–**5** and known compound JBIR-12.

In our previous report, the Yellow River wetland-derived *Talaromyces funiculosus* HPU-Y01 could produce a biologically active compound funitatin A by adding SAHA to the culture medium (Wang et al., 2022). To explore the metabolic potential of this fungus, we selected this strain for further chemical investigation by cultivating it with aniline instead of SAHA. As a result, HPLC-UV analysis showed obvious differences from the control fermentation (Supplementary Figure S1). Chemical investigation of crude extracts resulted in the discovery of a novel aniline-containing polyketide tanicutone A (**1**), two new bicyclic polyketides tanicutones B-C (**2**–**3**), and a new related trienoic acid 8-methyldeca-2,4,6-trienoic acid (**5**), together with a known compound **4** (Figure 1). Tanicutone B (**2**) has wide bacteriostatic activities (MIC values: 0.17 ~ 5.53 μg/mL). This article describes the fermentation, separation, structure identification, and biological activity assay.

## Materials and methods

### General experimental procedures

Optical rotations were obtained on a JASCOP-1020 digital polarimeter. UV spectra were recorded on Waters 2487, while the ECD spectrum was measured on the JASCO J-815 spectropolarimeter. IR spectra were taken on a Nicolet NEXUS 470 spectrophotometer as KBr disks. ^1^H NMR, ^13^C NMR, DEPT, and 2D NMR spectra were recorded on an Agilent 400 MHz DD2 spectrometer. HRESIMS data were obtained using a Thermo Scientific LTQ Orbitrap XL mass spectrometer. MPLC was performed using a C_18_ column (Agela Technologies, YMC-Pack ODS-A, 3 cm × 40 cm, 5 μm) at a flow rate of 20 mL/min. Preparative HPLC collection used a C_18_ column (Waters, YMC-Pack ODS-A, 10 mm × 250 mm, 5 μm, 3 mL/min). Aniline was purchased from Aladdin (Shanghai, China).

### Fungal material

The working stocks were preserved on potato dextrose agar slants stored at 4°C. The isolation and identification of *Talaromyces funiculosus* HPU-Y01 had been previously described (Wang et al., 2022).

### Culture, fermentation, and extraction

Erlenmeyer flasks (500 mL) containing 150 mL fermentation media were directly inoculated with spores. The media contained glucose (40 g) and peptone (10 g) dissolved in 1 L tap water in the presence of 300 μM aniline. The flasks were cultured at 28°C on a rotary platform shaker at 180 rpm for 7 days. The fermentation broth (30.0 L) was filtered through cheesecloth to separate the supernatant from the mycelia. The supernatant was extracted with EtOAc (3 × 30.0 L) and evaporated under reduced pressure to give 3.5 g extract.

### Purification

The extract was separated by MPLC (MeOH: H_2_O, 30–100%, 60 min) to give five fractions (Fractions 1–5). Fraction 2 was separated by semi-preparative HPLC eluting with MeCN-H_2_O (48:52) to obtain compound **5** (14.0 mg, *t*_R_ = 18.0 min), and compound **4** (40.0 mg, *t*_R_ = 22.0 min). Fraction **3** was separated by semi-preparative HPLC eluting with MeCN-H_2_O (55:45) to obtain compound **3** (12.0 mg, *t*_R_ = 16.0 min) and compound **2** (13.0 mg, *t*_R_ = 24.0 min). Fraction 4 was separated by semi-preparative HPLC eluting with MeCN-H_2_O (65:35) to obtain compound **1** (16.0 mg, *t*_R_ = 25.0 min).

Tanicutone A (**1**): pale yellow oil, [*α*]^20^_D_ -108.0 (*c* 0.10, MeOH); ECD (MeOH) *λ*_max_ (∆ε) 240 (+11), 290 (+17), 320 (+12), and 360 (−20) nm; ^1^H and ^13^C NMR data, see Tables 1, 2 and Supplementary Figures S2–S7; HRESIMS *m/z* 484.2128 [M−H]^−^ (calcd for C_30_H_30_NO_5_, 484.2129; Supplementary Figure S8); UV (MeOH) *λ*_max_ (log *ε*): 264 (1.40), 310 (4.66), and 382 (1.38) nm (Supplementary Figure S9); IR *ν*_max_ (KBr): 3,438, 2,960, 1,635, 1,596, 1,438, 1,155, 1,054, and 1,004 cm^−1^ (Supplementary Figure S10).

**Table 1:** ^1^H (400 MHz) NMR Data for Compounds 1–5 in DMSO-*d*_6_.

No.	**1**	**2**	**3**	**4**	**5**
7	6.52, d (2.0)	-			-
9	7.14, d (2.0)	7.39, s	7.12, s	7.18, s	-
11	8.51, d (12.7)	1.51, s	1.45, s	1.48, s	-
12	1.47, s	1.62, s	1.44, s	1.48, s	-
13	2.43, s	10.17, s	2.30, s	4.88, d (12.1), 4.64, d (11.7)	-
2′	6.08, d (15.2)	6.08, d (15.2)	6.07, d (15.2)	6.07, d (15.2)	5.84, d (15.2)
3′	7.26, dd (15.2, 11.3)	7.33, dd (15.2, 11.3)	7.31, dd (15.2, 11.3)	7.32, dd (15.2, 11.3)	7.19, dd (15.1, 11.3)
4′	6.42, dd (15.0, 11.3)	6.42, dd (15.0, 11.3)	6.42, dd (15.0, 11.3)	6.42, dd (15.0, 11.3)	6.35, dd (14.9, 11.3)
5′	6.74, dd (14.9, 10.8)	6.78, dd (14.9, 10.8)	6.76, dd (14.9, 10.8)	6.76, dd (14.9, 10.8)	6.66, dd (14.9, 10.8)
6′	6.19, dd (15.3, 10.8)	6.20, dd (15.3, 10.8)	6.20, dd (15.3, 10.8)	6.20, dd (15.3, 10.8)	6.15, dd (15.3, 10.8)
7′	5.91, dd (15.3, 7.7)	5.92, dd (15.3, 7.7)	5.92, dd (15.3, 7.7)	5.92, dd (15.3, 7.7)	5.86, dd (15.2, 7.8)
8′	2.16, m	2.16, m	2.16, m	2.16, m	2.13, m
9′	1.34, m	1.33, m	1.34, m	1.34, m	1.33, m
10′	0.84, t (7.3)	0.83, t (7.3)	0.83, t (7.3)	0.84, t (7.3)	0.83, t (7.3)
11′	0.99, d (6.7)	0.99, d (6.7)	0.99, d (6.7)	0.99, d (6.7)	0.98, d (6.7)
2″	7.56, d (7.8)	-	-	-	-
3″	7.41, t (7.5)	-	-	-	-
4″	7.18, t (7.4)	-	-	-	-
5″	7.41, t (7.5)	-	-	-	-
6″	7.56, d (7.8)	-	-	-	-
7-OH	-	-	8.75, s	9.18, s	-
8-OH	10.37, s	-	10.61, s	10.52, s	-
-NH	12.51, d (12.6)	-	-	-	-

**Table 2:** ^13^C (100 MHz) NMR data for compounds **1**–**5** in DMSO-*d*_6_.

No.	**1** *δ*_C_, type	**2** *δ*_C_, type	**3** *δ*_C_, type	**4** *δ*_C_, type	**5** *δ*_C_, type
1	101.8, C	73.5, C	74.1, C	74.2, C	-
2	191.9, C	203.7, C	204.8, C	204.5, C	-
3	84.1, C	83.6, C	84.1, C	84.1, C	-
4	192.4, C	190.5, C	191.1, C	191.5, C	-
5	116.9, C	120.1, C	119.8, C	119.8, C	-
6	143.8, C	120.6, C	125.7, C	127.6, C	-
7	116.1, CH	148.8, C	143.5, C	144.3, C	-
8	161.8, C	152.4, C	150.2, C	150.9, C	-
9	105.4, CH	116.1, CH	110.1, CH	111.4, CH	-
10	141.2, C	139.5, C	138.8, C	138.9, C	-
11	145.4, CH	31.1, CH_3_	32.8, CH_3_	32.8, CH_3_	-
12	23.2, CH_3_	23.0, CH_3_	23.2, CH_3_	23.2, CH_3_	-
13	22.8, CH_3_	195.7, CH	12.9, CH_3_	55.5, CH_2_	-
1′	164.4, C	164.5, C	164.3, C	164.3, C	167.6, C
2′	118.9, CH	117.6, CH	118.2, CH	118.1, CH	120.9, CH
3′	145.9, CH	147.1, CH	146.6, CH	146.6, CH	144.4, CH
4′	128.0, CH	127.8, CH	127.9, CH	127.9, CH	128.1, CH
5′	142.1, CH	142.9, CH	142.5, CH	142.5, CH	140.9, CH
6′	128.3, CH	128.3, CH	128.3, CH	128.3, CH	128.3, CH
7′	146.2, CH	146.7, CH	146.5, CH	146.5, CH	145.4, CH
8′	38.0, CH	38.0, CH	38.0, CH	38.0, CH	38.0, CH
9′	28.9, CH_2_	28.8, CH_2_	28.8, CH_2_	28.8, CH_2_	28.9, CH_2_
10′	11.6, CH_3_	11.6, CH_3_	11.6, CH_3_	11.5, CH_3_	11.5, CH_3_
11′	19.5, CH_3_	19.4, CH_3_	19.4, CH_3_	19.4, CH_3_	19.5, CH_3_
1″	139.2, C	-	-	-	-
2″	117.7, CH	-	-	-	-
3″	129.6, CH	-	-	-	-
4″	124.7, CH	-	-	-	-
5″	129.6, CH	-	-	-	-
6″	117.7, CH	-	-	-	-

Tanicutone B (**2**): pale yellow oil, [*α*]^20^_D_ -93.0 (*c* 0.10, MeOH); ECD (MeOH) *λ*_max_ (∆ε) 245 (+15), 290 (−15), 315 (+30), and 356 (−16) nm; ^1^H and ^13^C NMR data, Tables 1, 2 and Supplementary Figures S11–S17; HRESIMS *m/z* 441.1554 [M−H]^−^ (calcd for C_24_H_25_O_8_, 441.1555; Supplementary Figure S18); UV (MeOH) *λ*_max_ (log *ε*): 245 (1.53), 306 (4.15) nm (Supplementary Figure S19); IR *ν*_max_ (KBr): 3,419, 2,962, 1,693, 1,610, 1,454, 1,292, 1,133, 1,078, and 1,001 cm^−1^ (Supplementary Figure S20).

Tanicutone C (**3**): pale yellow oil, [*α*]^20^_D_ -120.0 (*c* 0.10, MeOH); ECD (MeOH) *λ*_max_ (∆ε) 245 (+45), 315 (+8), and 335 (−60) nm; ^1^H and ^13^C NMR data, Tables 1, 2 and Supplementary Figures S21–S27; HRESIMS *m/z* 427.1761 [M−H]^−^ (calcd for C_24_H_27_O_7_, 427.1762; Supplementary Figure S28); UV (MeOH) *λ*_max_ (log *ε*): 242 (1.28), 306 (4.88) nm (Supplementary Figure S29); IR *ν*_max_ (KBr): 3,427, 2,960, 1,695, 1,616, 1,302, 1,136, 1,091, and 1,007 cm^−1^ (Supplementary Figure S30).

Compound **4**: pale yellow oil, [*α*]^20^_D_ -175.7 (*c* 0.10, MeOH); ECD (MeOH) *λ*_max_ (∆ε) 245 (+60), 290 (+20), and 335 (−85) nm; ^1^H and ^13^C NMR data, Tables 1, 2 and Supplementary Figures S31–S37; HRESIMS *m/z* 443.1712 [M−H]^−^ (calcd for C_24_H_27_O_8_, 443.1711; Supplementary Figure S38); UV (MeOH) *λ*_max_ (log *ε*): 244 (1.30), 313 (4.80) nm (Supplementary Figure S39); IR *ν*_max_ (KBr): 3,419, 2,958, 1,680, 1,608, 1,298, 1,140, 1,086, and 1,005 cm^−1^ (Supplementary Figure S40).

8-methyldeca-2,4,6-trienoic acid (**5**): pale yellow oil, [*α*]^20^_D_ -60.0 (*c* 0.10, MeOH); ECD (MeOH) *λ*_max_ (∆ε) 245 (+20), 280 (+15), 300 (−5), and 330 (−8) nm; ^1^H and ^13^C NMR data, Tables 1, 2 and Supplementary Figures S43–S49; HRESIMS *m/z* 181.1221 [M + H]^+^ (calcd for C_11_H_17_O_2_, 181.1223; Supplementary Figure S50); UV (MeOH) *λ*_max_ (log *ε*): 301 (3.42) nm (Supplementary Figure S51); IR *ν*_max_ (KBr): 3,400, 2,962, 2,920, 1,701, 1,240, and 995 cm^−1^ (Supplementary Figure S52).

### Bioactivity assay

The antimicrobial activities of **1**–**5** against *Vibrio parahaemolyticus*, *Proteus species*, *Escherichia coli*, *Bacillus subtilis*, Methicillin-Resistant *Staphylococcus aureus*, and *Pseudomonas adaceae* were evaluated as previously reported by using the agar dilution method (Andrews, 2001; Yu et al., 2019). All experiments were performed in triplicate, and ciprofloxacin was used as a positive control. All strains were donated by Qingdao Municipal Hospital.

### Computation section

Conformational searches were run by employing the “systematic” procedure implemented in Spartan′ 14 using Merck molecular force field (MMFF). All MMFF minima were reoptimized with DFT calculations at the B3LYP/6–31 + G(d) level using the Gaussian09 program (Frisch et al., 2010). The geometry was optimized starting from various initial conformations, with vibrational frequency calculations confirming the presence of minima. Time-dependent DFT calculations were performed on four lowest-energy conformations for (3*R*)-**1a**, three lowest-energy conformations for (1*R*, 3*R*)-**2a**, two lowest-energy conformations for (1*S*, 3*R*)-**2b**, three lowest-energy conformations for (1*R*, 3*R*)-**3a**, three lowest-energy conformations for (1*S*, 3*R*)-**3b**, two lowest-energy conformations for (1*R*, 3*R*)-**4a**, two lowest-energy conformations for (1*S*, 3*R*)-**4b**, and four lowest-energy conformations for (8′*R*)-**5** (>3% population) using 30 excited states, and using a polarizable continuum model (PCM) for MeOH. ECD spectra were all generated using the program SpecDis (Bruhn et al., 2011) by applying a Gaussian band shape with 0.25 eV, from dipole length rotational strengths. The dipole velocity forms yielded negligible differences. The spectra of the conformers were combined using Boltzmann weighting, with the lowest-energy conformations accounting for about 97% of the weights. The calculated spectra were shifted by 0 nm for **1a/3a**/**3b**, 10 nm for **2a**/**2b/5**, and 15 nm for **4a**/**4b** to facilitate comparison to the experimental data. Detailed computational data have shown in Supplementary Figures S53–S60 and Supplementary Tables S1–S8.

## Results and discussion

### Structure elucidation of the new compounds

**Figure 2:**
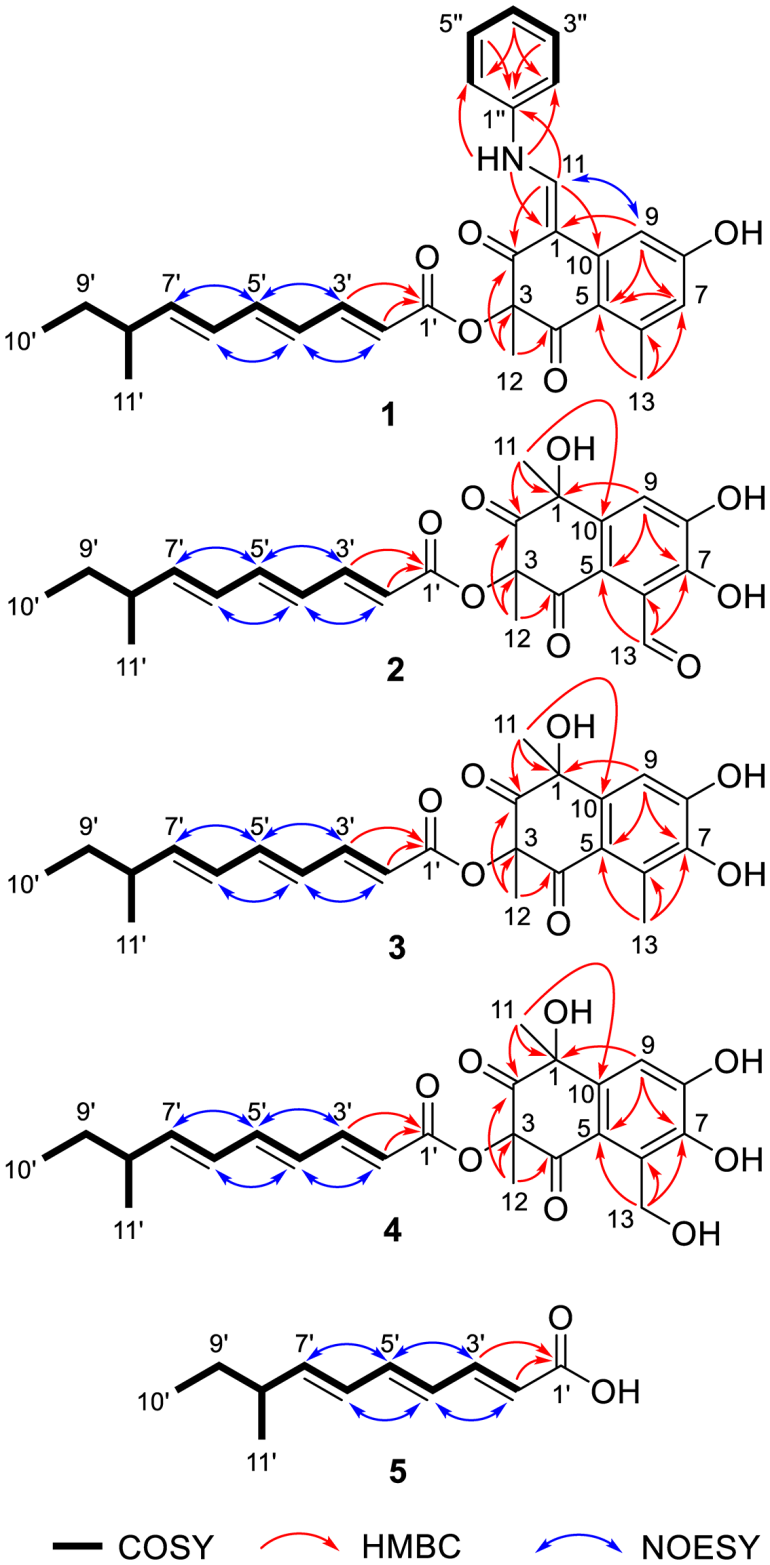
Key COSY, HMBC, and NOESY correlations of **1**–**5**.

Tanicutone A (**1**) was isolated as a pale-yellow oil. The molecular formula was determined as C_30_H_31_NO_5_ according to HRESIMS analysis, indicating 16 degrees of unsaturation. The 1D NMR data (Tables 1, 2) of **1** suggested the presence of 4 methyls, 1 methylene, 14 sp^2^ methines, 1 sp^3^ methine, and 10 non-protonated carbons including three carbonyls. The ester chain (from C-1′ to C-11′) was deduced from the COSY cross peaks (H-2′/H-3′/H-4′/H-5′/H-6′/H-7′/H-8′/H_2_-9′/H_3_-10′/H-8′/H_3_-11′) and key HMBC correlations from H-2′ and H-3′ to C-1′ (Figure 2). The tetrahydronaphthalene moiety was indicated by the HMBC correlations from H-7 to C-5 and C-9, from H-9 to C-1, C-5, and C-7, from H-11 to C-1, C-2, and C-10, from H_3_-12 to C-2, C-3, and C-4 and from H_3_-13 to C-5, C-6, and C-7, as well as the chemical shifts of C-2 (*δ*_C_ 191.9) and C-4 (*δ*_C_ 192.4). The spectroscopic data (Tables 1, 2) of these two fragments showed that they were linked by C3-O-C1′, which were similar to those of known compound JBIR-12 (Izumikawa et al., 2009; Figure 1) but differed by the substituted groups in the naphthalene ring. The substituent group in the C-6 was methyl of **1** not hydroxymethyl of JBIR-12, based on the chemical shifts of CH_3_-13 (*δ*_C_ 22.8, *δ*_H_ 2.43), which were also in agreement with the 2D NMR data (Figure 2). Another difference is that the H-7 of **1** was unsubstituted according to the chemical shifts of CH-7 (*δ*_C_ 116.1, *δ*_H_ 6.52, d, *J* = 2.0 Hz), which was supported by the HMBC correlations from H-7 to C-5 and C-9. Detailed analysis of the NMR data of **1** revealed **1** contained one extra aniline group compared with the known compound JBIR-12. Key HMBC correlations from NH to C-1 and C-2″ (C-6″) and from H-11 to C-2, C-10 and C-1″ further proved the presence of an aniline group and attached it to the C-11 atom.

Tanicutones B-C (**2–3**) were all isolated as pale-yellow oils. The ^1^H and ^13^C NMR data of **2**–**3** (Tables 1, 2) indicated that both of them possessed the same skeletons as JBIR-12, differing only by the substituting groups at C-6. The substituent group in the C-6 was aldehyde of **2** and methyl of **3**, which was based on the chemical shift of CH-13 (*δ*_C_ 195.7, *δ*_H_ 10.17) of **2** and CH_3_-13 (*δ*_C_ 12.9, *δ*_H_ 2.30) of **3**, together with HRESIMS data.

The NMR data of compound **4** (Tables 1, 2) are highly similar to those of JBIR-12 (Izumikawa et al., 2009). Then the ^1^H and ^13^C NMR data of compound **4** were remeasured in CD_3_OD instead of DMSO-*d*_6_ according to the literature. The results displayed that they should be the same compound (Δ*δ*_H_ < 0.02, Δ*δ*_C_ < 0.2; Supplementary Figures S41, S42). However, the relative and absolute configurations of the known compound JBIR-12, which was isolated from *Penicillium* sp., were not determined (Izumikawa et al., 2009).

Compound **5** was isolated as a pale-yellow oil with the molecular formula of C_11_H_16_O_2_ based on HRESIMS analysis. The 1D and 2D NMR data of **5** (Tables 1, 2; Figure 2) indicated that **5** possessed a similar triene chain with compounds **1**–**4**, differing only by the terminal carboxylic acid of **5** instead of the ester group of **1**–**4**.

The geometry of double bonds in the triene chain of **1**–**5** was deduced to be *E* by the NOESY correlations (H-2′/H-4′, H-3′/H-5′, H-4′/H-6′, and H-5′/H-7′; Figure 2). The geometry of double bond C1-C11 of **1** was deduced to be *Z* by the NOESY correlation (H-9/H-11; Figure 2).

**Figure 3:**
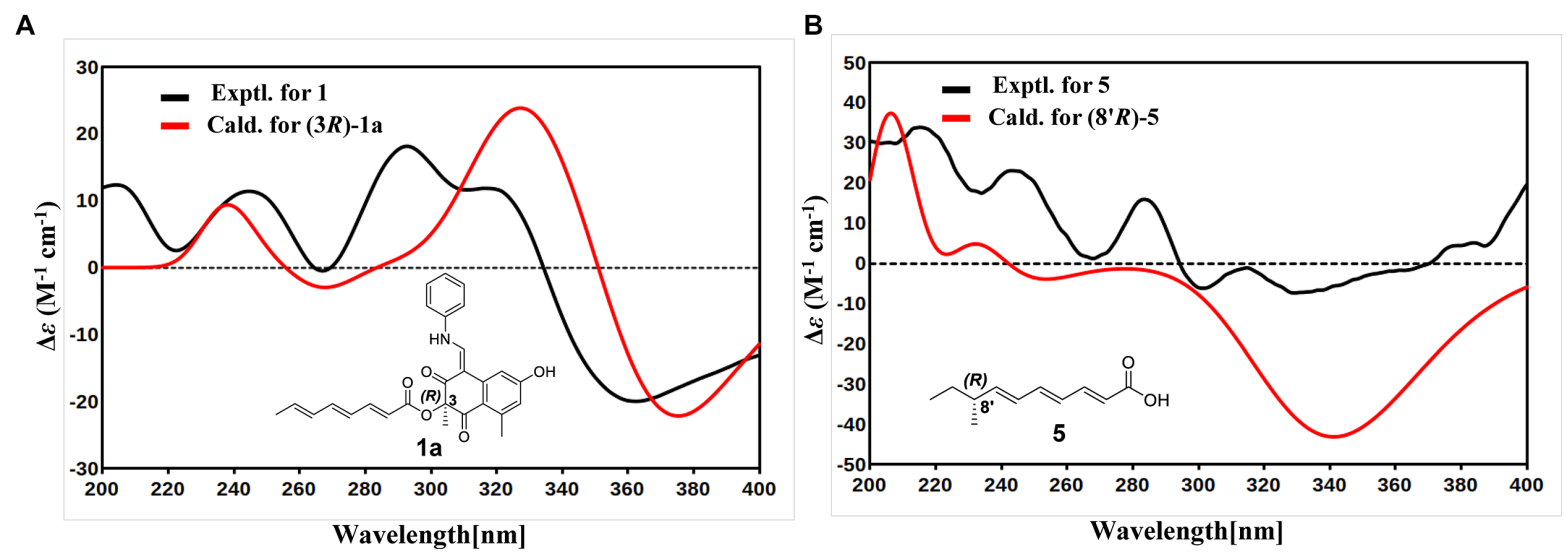
**(A)** Experimental ECD spectrum of 1 (black curve) and calculated ECD of (3*R*)-1a (red curve). **(B)** Experimental ECD spectrum of 5 (black curve) and calculated ECD of (8′*R*)-5 (red curve).

**Figure 4:**
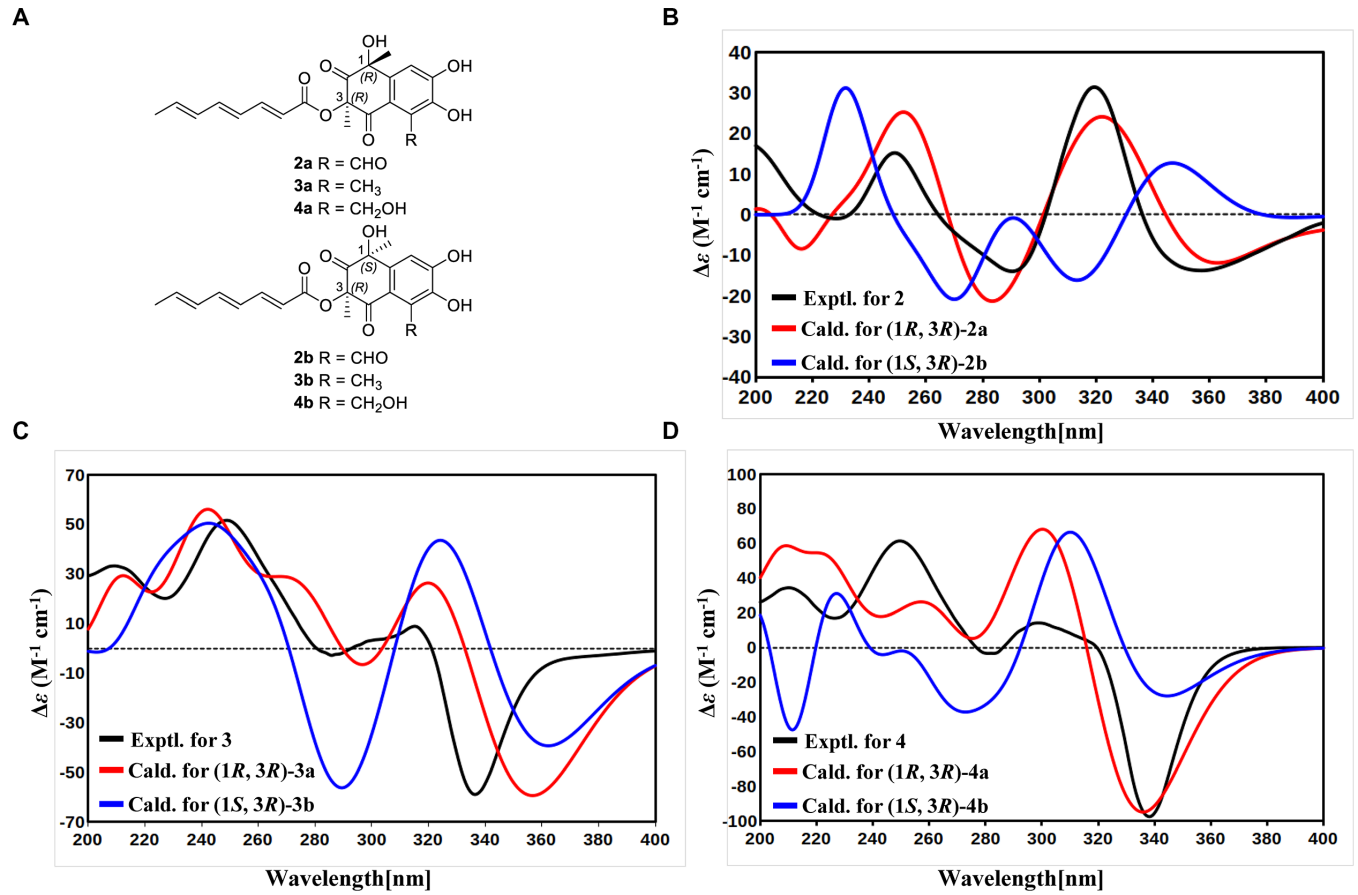
**(A)** Structures of truncated models 2a-4a and 2b-4b. **(B)** Experimental ECD spectrum of 2 (black curve), calculated ECD of (1*R*, 3*R*)-2a (red curve), and calculated ECD of (1*S*, 3*R*)-2b (blue curve). **(C)** Experimental ECD spectrum of 3 (black curve), calculated ECD of (1*R*, 3*R*)-3a (red curve), and calculated ECD of (1*S*, 3*R*)-3b (blue curve). **(D)** Experimental ECD spectrum of 4 (black curve), calculated ECD of (1*R*, 3*R*)-4a (red curve), and calculated ECD of (1*S*, 3*R*)-4b (blue curve).

With the consideration of the biogenetic origin and co-isolation of **1**–**5**, compounds **1**–**5** should have the same absolute configurations. The absolute configuration of C-3 was determined by comparing the experimental ECD curve of **1** with the one calculated from the truncated model (3*R*)-**1a** (Figure 3A). The good agreement between the calculated ECD spectra of(3*R*)-**1a** with the experimental result suggested the absolute configuration of **1** as 3*R* (Figure 3A). The relative stereo-relationship of the two chiral centers (C-1 and C-3) of compound **2** was not established due to the lack of valid signals. The computational ECD spectra of the two truncated models (1*R*, 3*R*)-**2a** and (1*S*, 3*R*)-**2b** (Figure 4A), covering all the possible absolute configurations, were calculated. Although calculated ECD curves of **2a** and **2b** are similar, the better agreement of the calculated ECD spectra of (1*R*, 3*R*)-**2a** (red curve) with the experimental data (black curve) of **2** suggest the 1*R*, 3*R* absolute configuration (Figure 4B). The absolute configurations of **3** and **4** were also established to be 1*R*, and 3*R* in the same way as **2** (Figures 4C,D). To determine the absolute configurations of C-8′, the solution conformers and ECD spectra of (8′*R*)-**5** were calculated. By comparing its experimental and calculated ECD curves, the absolute configuration of **5** was established to be 8′*R* (Figure 3B). Finally, the absolute configurations of all compounds were determined as (3*R*, 8′*R*)-**1**, (1*R*, 3*R*, 8′*R*)-**2**, (1*R*, 3*R*, 8′*R*)-**3**, and (1*R*, 3*R*, 8′*R*)-**4**.

### Bioactivity assay

The antimicrobial activities of **1**–**5** against *V. parahaemolyticus*, *P. species*, *E. coli*, *B. subtilis*, Methicillin-Resistant *S. aureus*, and *P. adaceae* were evaluated (Andrews, 2001; Yu et al., 2019). Compounds **2**–**5** showed broad inhibition against a panel of strains with MIC values ranging from 0.17 to 9.00 μg/mL, while compound **1** was inactive (Table 3). Compound **2** with aldehyde group in C-6 showed more activity than **3** and **4**, especially for the *V. parahaemolyticus* with an MIC value of 0.17 μg/mL (ciprofloxacin as a positive control, MIC = 0.13 μg/mL), indicating the aldehyde was important for antimicrobial activity.

**Table 3:** Antimicrobial assays of compounds **1**–**5** (MIC μg/mL).

No.	*V. parahaemolyticus*	*P. species*	*E. coli*	*B. subtilis*	*MRSA*	*P. adaceae*
**1**	>10.0	>10.0	>10.0	>10.0	>10.0	>10.0
**2**	0.17	0.34	0.34	0.69	1.38	5.53
**3**	5.35	2.68	2.68	2.68	>10.0	>10.0
**4**	5.55	1.39	2.78	>10.0	>10.0	>10.0
**5**	>10.0	2.25	2.25	1.13	9.00	>10.0
CIP^a^	0.13	0.08	0.08	0.04	0.08	0.03

a Ciprofloxacin was used as a positive drug.

### Plausible biogenetic pathways proposed for 1–5

**Figure 5:**
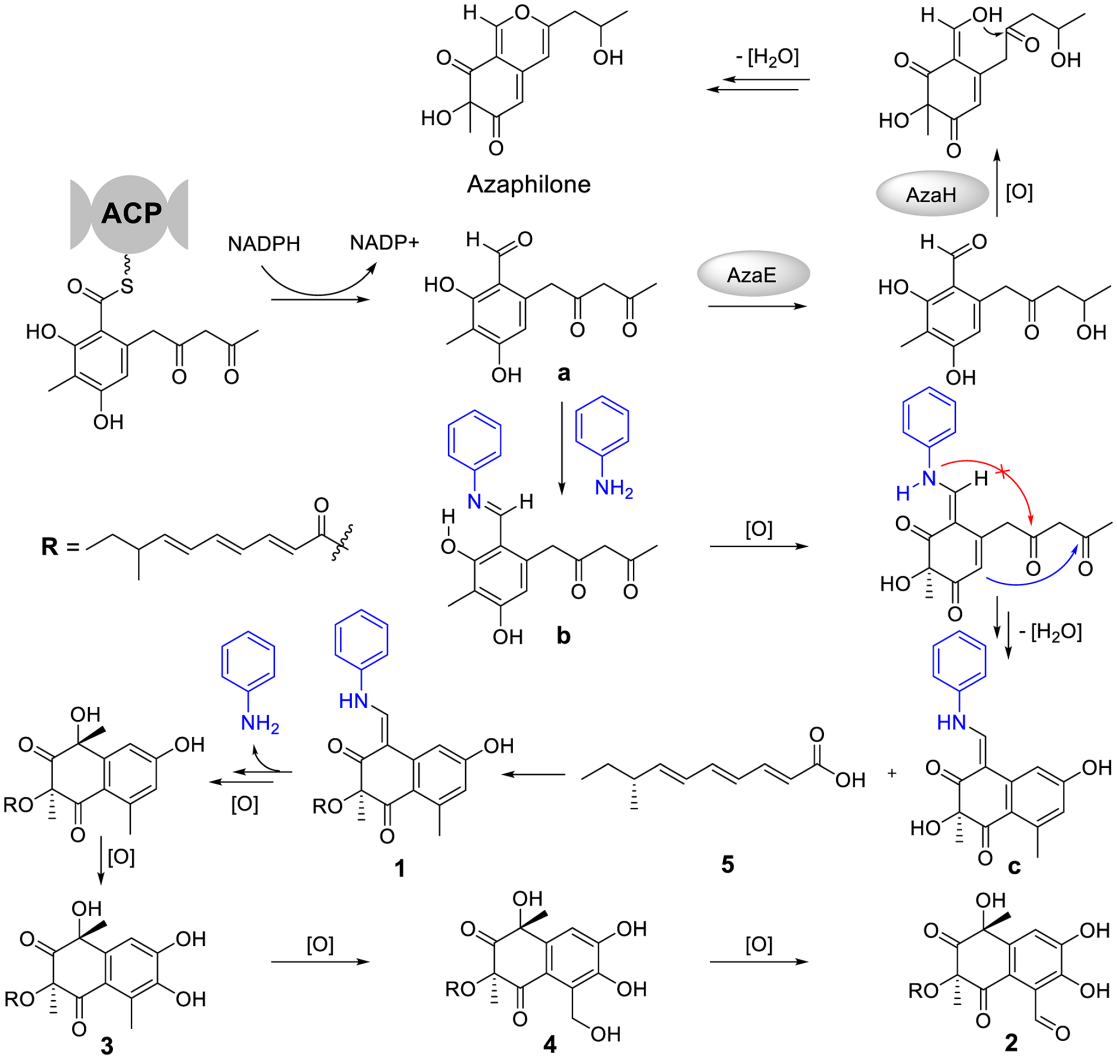
Plausible formation process of compounds **1**–**5**.

The special aniline-induced production of **1**–**5** triggered off our interest in their formation routes. Detailed analysis of the structures of **1**–**4** unveiled that their basic structure was probably assembled from a key polyketide unit **a** (Figure 5), which was a precursor to form azaphiliones (a common class of fungal metabolites characterized by a highly oxygenated pyrano-quinone bicyclic core) (Zabala et al., 2012; Gao et al., 2013). We propose that the addition of aniline could intercept the aldehyde intermediate **a** to form the Schiff base **b** (Adpressa et al., 2017), and then blocked the formation of azaphiliones. Further oxidation at C-3 of **b** and intramolecular nucleophilic addition (shown as the blue arrow, not the red arrow which was the way to form azaphiliones) afforded the core skeleton **c**. Then, **1** was generated by intermolecular dehydration involving 8-methyldeca-2,4,6-trienoic acid (**5**) with **c**. Finally, the removal of aniline moiety and further oxidation of **1** resulted in the formation of **2**–**4**.

## Conclusion

In summary, one new aniline-containing polyketide tanicutone A (**1**), three new bicyclic polyketides tanicutones B-C (**2**–**3**), a new unsaturated fatty acid **5,** and a known compound **4** were obtained from the Yellow River wetland-derived fungus *T. funiculosus* by adding aniline. The absolute configurations of **1**–**5** were determined by calculating ECD. Compound **2** containing a highly reactive aldehyde group exhibited the best antimicrobial activity against *V. parahaemolyticus* with an MIC value of 0.17 μg/mL. Our findings indicate that small molecule aniline could be an effective tool in exploiting the metabolic potential of fungi by interfering with conventional biosynthetic pathways.

## Data availability statement

The original contributions presented in the study are included in the article/supplementary material; further inquiries can be directed to the corresponding authors.

## Author contributions

ZW designed the experiments, prepared the manuscript, and was involved in the isolation of compounds. CN, LY, and YZ performed strain fermentation and extraction. DL contributed to the bioactivity assay. ZL and GW contributed to determining the structures of isolated compounds. ZZ and XH supervised the work and revised the manuscript. All authors contributed to the article and approved the submitted version.

## Funding

This study was financially supported by the National Natural Science Foundation of China (22207030), the Natural Science Foundation of Henan (222301420095), the 2022 Innovative scientific research team of Henan Polytechnic University (T2022-3), the Key Scientific and Technological Project of Henan Science and Technology Department (212102311020 and 232102310393), the Fundamental Research Funds for the Universities of Henan Province (NSFRF200334, NSFRF230629, and NSFRF230633), the Young Key Teachers Funding Program Project of Henan Polytechnic University (2020XQG-20), and Patent Implementation License Project (H22-554).

## Conflict of interest

The authors declare that the research was conducted in the absence of any commercial or financial relationships that could be construed as a potential conflict of interest.

## Publisher’s note

All claims expressed in this article are solely those of the authors and do not necessarily represent those of their affiliated organizations, or those of the publisher, the editors and the reviewers. Any product that may be evaluated in this article, or claim that may be made by its manufacturer, is not guaranteed or endorsed by the publisher.
